# Multilayered Nanocomposites Prepared through Quadruple-Layering Approach towards Enhanced Mechanical Performance

**DOI:** 10.3390/molecules27154852

**Published:** 2022-07-29

**Authors:** Yingji Wu, Haoran Ye, Guiyang Zheng, Changtong Mei, Liping Cai, Quyet Van Le, Changlei Xia

**Affiliations:** 1 Jiangsu Co-Innovation Center of Efficient Processing and Utilization of Forest Resources, International Innovation Center for Forest Chemicals and Materials, College of Materials Science and Engineering, Nanjing Forestry University, Nanjing 210037, Jiangsu, China; wuyingji@njfu.edu.cn (Y.W.); yehaoran@njfu.edu.cn (H.Y.); nanlinguiyangzheng@njfu.edu.cn (G.Z.); lipingcai@gmail.com (L.C.); 2 Department of Materials Science and Engineering, Institute of Green Manufacturing Technology, Korea University, 145 Anam-ro, Seongbuk-gu, Seoul 02841, Korea; quyetbk88@korea.ac.kr; 3 DeHua TB New Decoration Materials Co., Ltd., Huzhou 313200, Zhejiang, China

**Keywords:** multilayered structure, nanoparticles, nanocomposites, mechanical properties

## Abstract

Multilayered materials are widely studied due to their special structures and great properties, such as their mechanical ones. In this paper a novel and effective technique, a quadruple-layering approach, was used to fabricate multilayered materials. This approach increases the number of layers rapidly via simple operations. Materials with 4, 16, and 64 layers with alternating layers of polypropylene and nanocomposites were fabricated using this approach, and their film morphology and mechanical properties were studied. The influence of the number of layers on the mechanical properties of the materials and the relationship between the mechanical properties of each material were investigated. The results illustrated that the tensile modulus and strength were enhanced and elongation at the break increased when the layer numbers of the multilayered materials increased. However, this approach has a defect in that as the layer number increases, the layer thickness was not uniform, thus restricting the improvement of properties. This may need to be further studied in future work.

## 1. Introduction

Multilayered structures are very common in nature, such as in trees [1], bones [2], butterfly wings [3], and nacre [4]. Bio-mimic multilayered materials can improve material properties such as mechanical [5,6], optical [7], electric [8], gas barrier [9], sound insulation [10], and fire retardant ones [11]. Multilayered materials are fabricated using different multilayering approaches including layer-by-layer [12], coextrusion [13], chemical vapor deposition coating [14], spray [15], and electrospinning [16], which have attracted great attention due to their structure features and property advantages. Multilayered co-extrusion is a technique that combines two or more different polymers or polymer composites into micro- or nano-layer materials with up to thousands of layers [17]. Co-extrusion, which includes two extruders and several layers of multipledies, doubles layer numbers when materials go through eachdie, and finally forms alternating multilayers with two or more different polymeric materials [18]. This technique operates at the melting state of polymers and composites, does not require any solvent, and is mainly used on thermoplastics [19]. However, this technique requires complex instruments and a huge amount of the materials; thus, the cost of material processing is massive.

Recently, many types of multilayered bio-composites have been developed to be applied as functional materials, such as bio-medical materials [20] and environmental protection products [21]. The properties of the developed products can be easily tuned by changing the deposition conditions. Wang, et al. [22] fabricated the Al/Ni multilayered composites with extremely exothermic reactions and great plasticity using the electro-deposition and hot-pressing technologies. The experiments indicated that as the time duration of hot-pressing increased, the bending displacement of the Al/Ni multilayered composite improved first and then declined sharply. Alvaredo-Atienza, et al. [23] fabricated polyetheretherketone (PEEK)/polyetherimide (PEI) multilayered composites by alternating PEEK/PEI layer stacking to avoid the complete miscibility of both polymers, keeping PEEK layers and PEI layers un-mixed along the thickness direction, as well as accelerating the formation of a smooth interfacial layer between PEEK and PEI layers. The results revealed that although the interface between the two pristine polymers remained unchanged after the long processing time, the mechanical properties including the Young modulus, tensile strength, and strain at break of the PEEK/PEI multilayered films were improved compared to the original PEEK and PEI films.

Multilayered films with clay and polyethylenimine bilayers fabricated using layer-by-layer assembly techniques exhibit a high hardness and a large elastic modulus [24]. These layer-by-layer films are different from the blended composites in that layer-by-layer materials’ clay formed separate layers from the polymers, while composites are mixtures of polymers and clay in the nanoscale. Clay additives being fully exfoliated in composites increases the modulus and strength of materials and are commonly used to increase mechanical properties of polymers [25]. For high-clay-content composites prepared using solvent evaporation assembly, the clay-forming nacre-like layered structure dramatically increases the tensile modulus and strength, with a clay content of 70 wt% the Young’s modulus and strength of the composite being 19.3 GPa and 219 MPa, which are 57 times and 5.5 times that of pure PVA, respectively [26]. However, the elongation at the break decreases rapidly with more than a 15 vol% of clay content, negatively affecting the application of the materials [26,27].

It has been reported that the multilayer technique can increase the tensile modulus without compensating the elongation at the break by fabricating the multilayered materials using alternating layers of pure low-density polyethylene and clay-filled composites [10]. Mazerolles, et al. [28] developed thermoplastic starch/polyethylene multilayered films for mechanical property testing, and the results revealed that the elongation at the break of clay-filled multilayered films dropped by around 30%, while the maximum tensile stress dropped by a factor of 2 compared to unfilled multilayered films. Clay-filled composite layers increased the strength of the materials and unfilled pure polymer layers by keeping the elongation at break values. For these multilayered materials, clay was mixed with polymers, forming composites layers which covered up the problem of inorganic materials not being stretchable and flexible [29]. There are also other factors that affect the properties of multilayered materials. Multilayering two different polymeric materials may change the crystalline structure of the polymers and nanoparticle dispersion in the filled layers as the layer thickness decreases, improving the film’s properties [6,18]. Moreover, the nanoparticles tend to form an ordered structure during the multilayering approach because it needs to compress or stretch the materials several times, and the nanoparticles prefer to arrange in a direction parallel to the film surface [30].

In this study, a new approach to multilayering with alternating layer arrangement is used to fabricate polypropylene (PP) and montmorillonite (MMT)/maleic anhydride-grafted PP (PPMA) composites. As an initially proposed technique by our group, this approach uses quadruple layer numbers in each manufacturing element, which needs to be cut and recombined into films under melting conditions using a melt press instrument. The advantage of this method is that the layer number increases rapidly in several cycles using a very basic instrument. The 4-layer (4L), 16-layer (16L), and 64-layer (64L) alternating multilayered materials are fabricated and their morphological and mechanical properties are examined.

## 2. Results and Discussion

**Figure 1:**
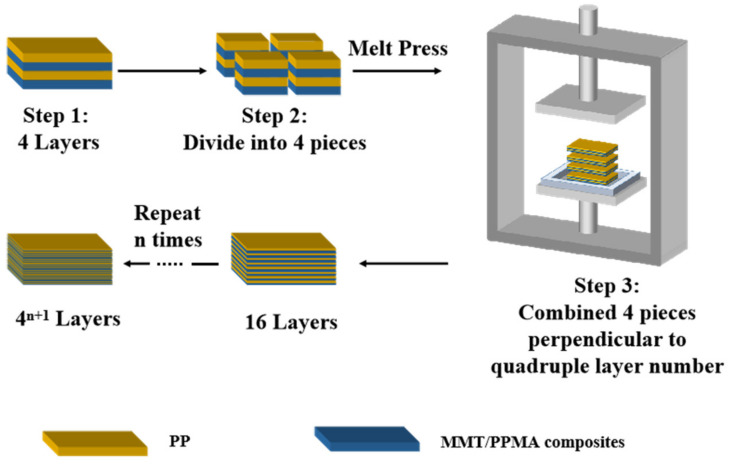
Scheme of multilayering process.

The multilayered materials were fabricated using a quadruple-layering approach, which was firstly developed by our group. The detail of the approach is described in the materials and methods section and Figure 1. This approach was applied to fabricate alternating layers of MMT filled and unfilled materials in the melt state of polymers using a melt press instrument to form 4 (4L), 16 (16L), and 64 layers (64L) of alternating multilayered materials.

**Figure 2:**
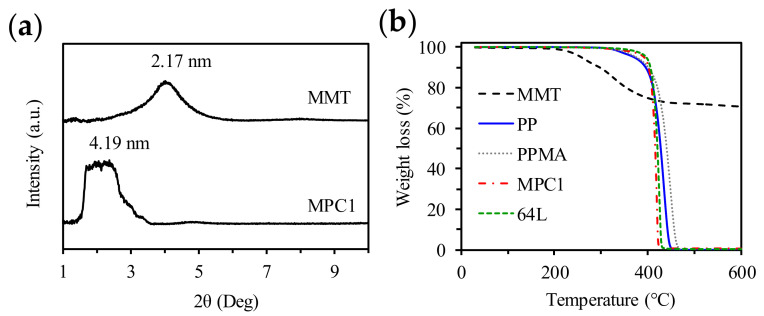
(**a**) XRD patterns of MMT and MPC1; (**b**) TGA of MMT, pure polymers, MPC1, and 64L.

MMT and PPMA blending composites were fabricated with MMT content 0.5 wt% (MPC0.5), 1 wt% (MPC1), and 2 wt% (MPC2) in the polymer matrices. XRD was used to evaluate the d-spacing between the MMT layers and the dispersion of MMT in the PPMA matrix. Figure 2a presents the XRD patterns of MMT and MPC1. The MMT used in the study was dimethyl diakyl amine-modified MMT. The (001) reflection of MMT was located at 2θ = 4.1°, which was a 2.17 nm d-spacing. The MPC1 exhibited broad reflection at around 2θ = 2.1°, with corresponding d-spacing of 4.19 nm. The results clearly show that the peak shifted to a lower angle, which indicated the intercalation of the polymers to the MMT layers.

The TGA of the polymers, MMT, composites and 64L multilayered material are shown in Figure 2b. MMT lost 29.1% weight after heating to 600 °C in a nitrogen atmosphere. MPC1 with 1% MMT and 64L multilayered material with 1% MMT in half of the layers have the residual weights of 0.67% and 0.36%, respectively. The degradation temperature of modified MMT is 257 °C, which is above the extruding and melt press temperatures. The degradation temperatures of the PPMA and MPC1 are 375 °C and 392 °C, respectively. The degradation temperature of MPC1 is higher than the pure PPMA, which indicates that the degradation temperature was increased by adding a small amount of MMT into the polymer. The degradation temperature of the 64L multilayered material is 396 °C, which is similar to MPC1 although the other alternating layer PP has a low degradation temperature (370 °C).

**Figure 3:**
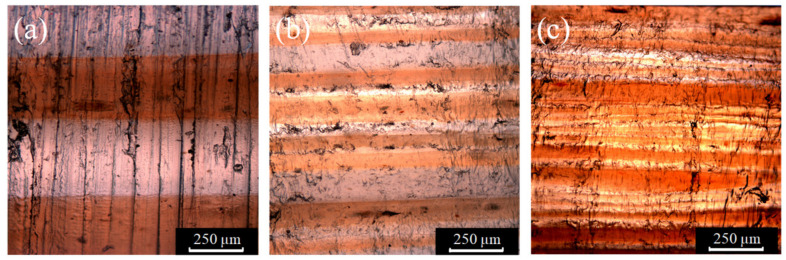
Optical and images of (**a**) 4-layer (4L), (**b**) 16-layer (16L), and (**c**) 64-layer (64L).

The layered structures are clearly seen in the images. Figure 3a–c show the optical images of the cross-sections of 4L, 16L, and 64L multilayered materials, respectively. The multilayered materials consist of alternating layers of MPC1 and pure PP. The MPC1 layers have a dark brown color, while the PP layers have a light brown color. The 4L multilayered material shows even layers with straight layer interface lines, and the layer thicknesses of PP and MPC1 are about 1:1. As the number of layers increases, the layer thicknesses are not as uniform as the 4L multilayered materials. For the 16L multilayered material, the layer interface lines are still straight; however, the layer thickness is not uniform. The 64L multilayered material shows the bending interlines with the ununiformed layer thickness; even the interface lines between layers disappear and the combination of layers can be observed. These problems are caused by the viscosity difference between the two materials and the non-uniform temperature during the process.

**Figure 4:**
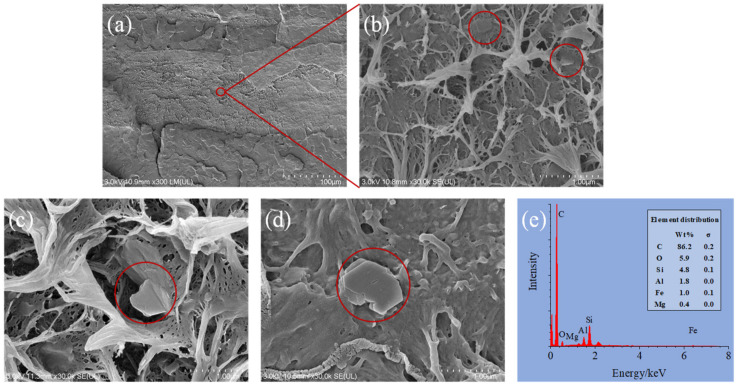
SEM images of 64L multilayered materials at (**a**) 300 magnification, (**b**–**d**) 30,000 magnification in different area with MMT nanoparticles highlighted with red circles, and (**e**) SEM-EDS spectra of multilayered materials.

Highly magnified SEM images show the nanoparticles in the filled layers. Figure 4a shows the 64L multilayered materials with filled (rough surface) and unfilled (smooth surface) layers. Magnifying the filled layers, the MMT nanoparticles can be seen highlighted with red circle in Figure 4b–d. The MMTs are imbedded in, half imbedded in, or totally revealed on the cross section. The size of the nanoparticles is about 300 nm to 1 µm. The MMT is dispersed in single pieces or as cluster in the PPMA. SEM-EDS analysis was carried out to detect the composition of the materials (Figure 4e). The multilayered material contains C, O, Si, Al, Fe, and Mg and the wt% is highly dependent on the area selected. Carbon composes more than 80%, which is from the polymer matrices, and the other elements consist of the clay composition [31].

**Figure 5:**
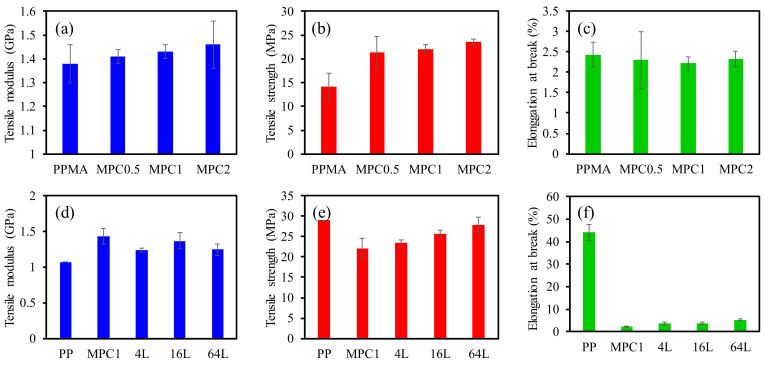
(**a**,**d**) tensile modulus, (**b**,**e**) tensile strength, (**c**,**f**) elongation at break of composites and multilayered materials.

**Figure 6:**
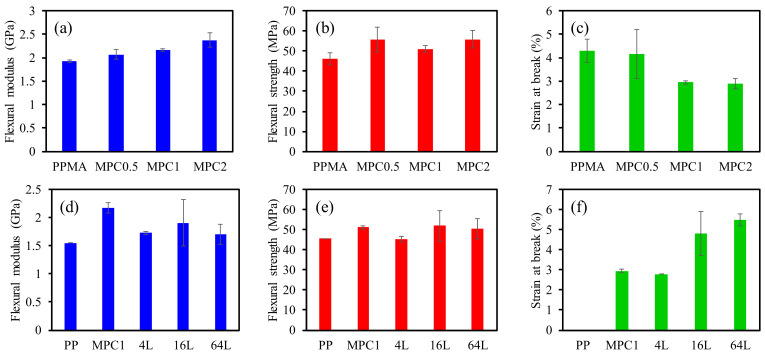
(**a**,**d**) flexural modulus, (**b**,**e**) flexural strength, and (**c**,**f**) strain at break of composites and multilayered materials.

The mechanical properties of the multilayers were evaluated by tensile and flexural measurements and the results are listed in Table 1, Figure 5 and Figure 6. Figure 5a–f shows the tensile modulus, tensile strength, and elongation at the break of pure polymers, composites, and multilayered materials. The tensile modulus of composites increased gradually upon increasing the clay content in the composites, as shown in Figure 5a. The tensile moduli of MPC0.5, MPC1, and MPC2 were increased by 2%, 4%, and 6% compared to the pure PPMA, respectively; however, the difference is not significant at the level of α = 0.05 (ANOVA test, *p*-value = 0.52, 0.35, and 0.44, respectively). Compared with that of the pure PMMA, the tensile strength exhibits a sudden increase of 50% after adding 0.5% MMT and maintains a slow increase of 56% and 65% for 1% and 2% of MMT composites, respectively. The tensile strength increases of the MPC0.5, MPC1, and MPC2 were significant at α = 0.05 compared to the pure PMMA (ANOVA test, *p*-value = 0.04, 0.01, and 0.01, respectively) The values of the elongation at the break of the composites did not display significant changes, however, exhibiting much lower values than the elongation at break values of the pure PP.

The tensile properties of the multilayered materials are shown in Figure 5d–f. MPC1 illustrates higher strength and lower flexibility compared to the pure PP, and the combination and multilayers of these two materials have tensile properties in between these two materials. The tensile modulus can be calculated based on Equation (1).

(1) $$E=E_{\mathrm{PP}}V_{\mathrm{PP}}+E_{\mathrm{MPC}}V_{\mathrm{MPC}}$$

where E is the tensile modulus of the whole multilayered material; E_PP_ and E_MPC_ are the tensile modulus of the PP layers and MPC1 layers, respectively; V_PP_ and V_MPC_ are the volume fractions of PP and MPC, respectively; E_PP_ and E_MPC_ values are 1.07 and 1.43 GPa, respectively. Both V_PP_ and V_MPC1_ values are 0.5 and the E value is 1.25 GPa according to the calculation of Eq. 1, which is similar to the E value tested. The tensile modulus of the 4L, 16L, and 64L multilayered materials is 1.24, 1.37, and 1.25 GPa, respectively. Elongation at break increases as the layer number increases. The elongation at break of MPC1, 4L, 16L, and 64L multilayered materials is 2.22%, 3.60%, 3.77%, and 5.21%, respectively. The elongation-at-break value increases since the alternating layers of PP have about 20 times higher elongation-at-break values compared to the MPC1. As the number of layers increases, each separate layer thickness decreases, causing increases in the PP and MPC1 surface contact areas.

The flexural measurement shows a similar trend to the tensile measurement (Figure 6a–f). Figure 6a shows the flexural modulus of PPMA and its composites. The flexural modulus of the composites increased gradually with the increase in MMT content. The strain-at-break value decreases as the MMT content increases. For the multilayered materials, the flexural modulus and strength improve with the increase in layer number and decrease again for the 64L multilayered material, because those observed layers are not uniform, as observed using the optical microscope. This causes property defects during the fabrication. The tensile strength and elongation-at-break value of the multilayered materials also increases with the increase in layer number.

Similar results were reported in certain recent studies, reporting that the filled multilayered materials showed better properties when compared with the single-layer composites, and properties increase as the layer numbers increase. Xia et al. [10] reported that tensile strength was not increased as the layer number increased; however, the elongation-at-break values increased obviously and the 128-layer sample had about 60% higher elongation-at-break values than the 8-layer ones. In this study, the tensile modulus increased slightly as the layer number increased, and the elongation-at-break values of the 64-layer sample were 44% higher than the 8-layer one. It has been proposed that nanoparticles tend to form an ordered direction in multilayered materials [10]. In this study, multilayered materials are fabricated multiple times under the melt press, which may cause the particle dispersion direction consistency, and as the layer thickness decreases, the particles may prefer the direction parallel to the film surface due to the restriction of layers. As the layer number increases, the particles may form a more ordered dispersion and cause the increase in mechanical properties.

## 3. Materials and Methods

### 3.1. Materials

Dimethyl diakyl amine-modified MMT was purchased from Sigma-Aldrich, Inc., Shanghai, China. PP with MFI 8.0 g/10 min at 230 °C was obtained from China Petroleum and Chemical Corp., Shanghai, China. PPMA with MFI 35–40 g/10 min at 230 °C and 1% maleic anhydride was purchased from Deba Polymer Co., Ltd., Nanjing, China

### 3.2. Preparation of the Nanocomposites

MMT and PPMA were dried in an oven for 10 h at 80 °C prior to compounding. Amounts of 0.5 wt%, 1 wt%, and 2 wt% MMT and PPMA were mixed at room temperature and followed by compounding with a twin-screw extruder at 190 °C and 20 rpm/min. The 0.5 wt% (MPC0.5), 1 wt% (MPC1), and 2 wt% (MPC2) MMT/PPMA composites were palletized for next step. The inorganic contents of MPC0.5, MPC1, and MPC2 are 0.35%, 0.7%, and 1.4%, respectively.

### 3.3. Preparation of Multilayers

MPC1 and PP were dried in the oven at 80 °C. The multilayered materials were prepared using the quadruple-layering approach, which is developed as shown in Figure 1. In Step 1, 1 mm MPC1 film and PP film were fabricated using a melt press separately and combined to form 4L alternating materials with a total film thickness of about 4 mm under a melting temperature of 180 °C and a pressure of 10 MPa/m^2^ for 5 min. In Step 2, the 4L film was cut into four equal-sized pieces perpendicularly across the surface. In Step 3, the four small 4L films were hot-pressed from 4 mm to 1 mm films under a melting temperature of 180 °C and a pressure of 0 MPa/m^2^ for 5 min, followed by a pressure of 10 MPa/m^2^ for another 5 min. The four pressed 4L films were combined perpendicularly to form a 16L film under a melting temperature of 180 °C and a pressure of 10 MPa/m^2^ for 5 min. Then, by repeating the same steps, a 64L film was achieved by combining four 16L films. Finally, the number of the layers become 4^n+1^, where n is the number of times the quadruple-layering approach was repeated. Here, 4L, 16L, and 64L multilayered materials were fabricated using the quadruple-layering approach.

### 3.4. Characterization

The dispersion of silicate layers of composites was observed by X-ray diffraction (XRD) measurements (D8 advance, Bruker, Germany) at room temperature. The XRD was scanning 2θ from 1° to 10° with a CuKα radiation (λ = 0.154 nm). The thermogravimetric analysis (TGA) was carried out using a TA TGA55 instrument at a heating rate of 10 °C/min from 30 °C to 600 °C under nitrogen gas atmosphere. The optical observation (Leica DM2500, Wetzlar, Germany) and scanning electron microscope (SEM) (Quanta 200, FEI company, Hillsboro, NH, USAand Hitachi Regulus 8100, Tokyo, Japan) were used to examine the morphology of the cross-section of the multilayered materials and nanoparticles. An energy-dispersive spectrometer (EDS) was used to analyze the composition of the materials. The tensile properties of the multilayered materials were determined using a universal testing machine (AGS-X, Shimadzu, Japan). Five samples were tested in each case and the average value was reported.

## 4. Conclusions

The multilayered materials were fabricated using the quadruple-layering approach. Optical and SEM images showed the layer structures and microstructures of the filled layers, indicating that the multilayered materials were successfully fabricated by alternating layers of PP and MMT filled composites using the quadruple-layering approach. The layers of 4L and 16L multilayered materials were quite uniform and showed straight layer interface line between the PP and composites layers. The 64L multilayered material illustrated visual layer disappearance and the layer combination of PP and composites layers. Tensile and flexural properties of the multilayered materials were improved compared to conventional composites. Tensile strength and elongation-at-break values increased with the increase in number of layers. The tensile modulus and flexural modulus of the 64L multilayered material slightly decreased compared to the 16L multilayered materials due to layer uniformity problems. Using this multilayering approach, multilayered composites can be fabricated with the melt press technique, achieving multilayered materials consisting of many layer numbers in several steps. However, this technique is still in development and needs further improvement to obtain more uniform layers as the layer number increases.

## Figures and Tables

**Table 1:** Summary of tensile and flexural properties of composites and multilayered materials.

Materials	Tensile Property			Flexural Property		
	Tensile Modulus (GPa)	Tensile Strength (MPa)	Elongation at Break (%)	Flexural Modulus (GPa)	Flexural Strength (MPa)	Strain at Break (%)
PP	1.07 ± 0.11	29.0 ± 2.4	44.2 ± 3.6	1.55 ± 0.09	45.6 ± 0.8	N/D
PPMA	1.38 ± 0.08	14.2 ± 2.8	2.4 ± 0.3	1.93 ± 0.03	46.0 ± 3.0	4.3 ± 0.5
MPC0.5	1.41 ± 0.03	21.3 ± 3.4	2.3 ± 0.7	2.07 ± 0.10	55.8 ± 6.2	4.2 ± 1.0
MPC1	1.43 ± 0.03	22.1 ± 0.8	2.2 ± 0.2	2.17 ± 0.03	51.1 ± 1.5	3.0 ± 0.1
MPC2	1.46 ± 0.10	23.5 ± 0.6	2.3 ± 0.2	2.38 ± 0.15	55.8 ± 4.4	2.9 ± 0.2
4L	1.24 ± 0.11	23.4 ± 0.9	3.6 ± 0.6	1.73 ± 0.41	45.2 ± 7.6	2.8 ± 0.1
16L	1.37 ± 0.08	25.7 ± 2.0	3.8 ± 0.5	1.91 ± 0.18	51.9 ± 4.9	4.8 ± 1.1
64L	1.25 ± 0.06	27.8 ± 1.2	5.2 ± 0.4	1.70 ± 0.02	50.6 ± 2.6	5.5 ± 0.3

## Data Availability

The data presented in this study are available in article.

## Sample Availability

Samples are available from the authors.
